# Gallic Acid Induces a Reactive Oxygen Species-Provoked c-Jun NH_2_-Terminal Kinase-Dependent Apoptosis in Lung Fibroblasts

**DOI:** 10.1155/2013/613950

**Published:** 2013-02-20

**Authors:** Chiu-Yuan Chen, Kun-Chieh Chen, Tsung-Ying Yang, Hsiang-Chun Liu, Shih-Lan Hsu

**Affiliations:** ^1^Graduate Institute of Natural Healing Sciences, Nanhua University, No. 55, Section 1, Nanhua Road, Zhongkeng, Dalin Township, Chiayi County 622, Taiwan; ^2^Division of Chest Medicine, Department of Internal Medicine, Taichung Veterans General Hospital, Taichung 407, Taiwan; ^3^Institute of Biomedical Science, National Chung Hsing University, Taichung 402, Taiwan; ^4^Department of Education and Research, Taichung Veterans General Hospital, No. 1650, Section 4, Taiwan Boulevard, Taichung 407, Taiwan; ^5^Department of Life Sciences, National Chung Hsing University, Taichung 402, Taiwan

## Abstract

Idiopathic pulmonary fibrosis is a chronic lung disorder characterized by fibroblasts proliferation and extracellular matrix accumulation. Induction of fibroblast apoptosis therefore plays a crucial role in the resolution of this disease. Gallic acid (3,4,5-trihydroxybenzoic acid), a common botanic phenolic compound, has been reported to induce apoptosis in tumor cell lines and renal fibroblasts. The present study was undertaken to examine the role of mitogen-activated protein kinases (MAPKs) in lung fibroblasts apoptosis induced by gallic acid. We found that treatment with gallic acid resulted in activation of c-Jun NH_2_-terminal kinase (JNK), extracellular signal-regulated kinase (ERK), and protein kinase B (PKB, Akt), but not p38MAPK, in mouse lung fibroblasts. Inhibition of JNK using pharmacologic inhibitor (SP600125) and genetic knockdown (JNK specific siRNA) significantly inhibited p53 accumulation, reduced PUMA and Fas expression, and abolished apoptosis induced by gallic acid. Moreover, treatment with antioxidants (vitamin C, N-acetyl cysteine, and catalase) effectively diminished gallic acid-induced hydrogen peroxide production, JNK and p53 activation, and cell death. These observations imply that gallic acid-mediated hydrogen peroxide formation acts as an initiator of JNK signaling pathways, leading to p53 activation and apoptosis in mouse lung fibroblasts.

## 1. Introduction

Idiopathic pulmonary fibrosis (IPF) is a progressive and usually fatal disorder with a reported median survival of 3 to 6 years from the time of diagnosis [1]. Clinically, IPF is characterized by the loss of lung epithelium and the formation of scar tissue within the lungs with accumulation of fibroblasts and myofibroblasts that deposit excessive extracellular matrix including collagen [2]. Increasing evidence shows that the abnormal wound repair process in response to alveolar epithelial injury is responsible for IPF and fibroblast-to-myofibroblast differentiation, which represents a key event during tissue repair [2]. The origin of pathological fibroblasts foci within the IPF lesion remains puzzling. Possibilities include differentiation of resident fibroblasts, recruitment of circulating fibroblast precursors, and transdifferentiation of epithelial cells into pathological fibroblast phenotypes [3, 4]. Apoptosis plays an important role in both normal lung homeostasis and lung remodeling associated with fibrotic lung disease. In IPF, widespread epithelial apoptosis is observed. In contrast to epithelial cells, fibroblasts derived from IPF lungs are more resistant to apoptosis than normal lung fibroblasts [5]. Whether apoptosis promotes or inhibits the pathogenesis of pulmonary fibrosis depends upon the cell type involved and the microenvironment of the affected lung. Immoderate cell loss in the alveolar epithelium may be important early in IPF progression, while reduced fibroblasts/myofibroblasts apoptosis has been associated with the formation of fibrotic lesions [6]. As such, novel therapies based on the stimulation of apoptosis of activated fibroblasts may prove beneficial to the treatment of patients with IPF.

Gallic acid (3,4,5-trihydroxybenzoic acid), a natural botanic phenolic compound, is widely distributed in green tea, red wine, and grapes, and so forth. Preclinical studies have shown that gallic acid possesses a variety of pharmacological activities, including antioxidant, anti-inflammatory, antimicrobial, and anticancer activities [7–9]. Recently, gallic acid has been found to exert potent antiviral effect at the therapeutic range of 0.8–0.05 *μ*g/mL [10]. In animal models, gallic acid reduces oxidative stress and enhances the levels of glutathione (GSH), GSH peroxidase, GSH reductase, and GSH *S*-transferase in hepatic tissue, as well as catalase in serum [11]. It also can inhibit the saturation of odd-chain polyunsaturated fatty acid [12] and has antiangiogenesis activities [13]. Exposure of human stomach cancer KATO III cells and human colon adenocarcinoma COLO 205 cells to gallic acid led to both growth inhibition and induction of apoptosis [14]. Hsu et al. reported that gallic acid induces apoptosis in preadipocyte cells via a Fas- and mitochondrial-mediated pathway [15]. Our previous report demonstrated that gallic acid induces apoptosis of mouse lung fibroblasts via a reactive-oxygen-species-(ROS-) dependent ataxia-telangiectasia-mutated-(ATM-) p53 activation pathway [16]. It is well known that excessive levels of intracellular ROS not only directly damage cells by oxidizing DNA, protein, and lipid, but also indirectly damage cells by activating a variety of stress-sensitive intracellular signaling pathways such as p38MAPK and JNK [17, 18]. Therefore, in this study, we attempted to address whether gallic acid-mediated ROS production can activate JNK and lead to apoptosis in mouse lung fibroblasts.

## 2. Material and Methods

### 2.1. Chemicals

Gallic acid, ascorbic acid (ASC), N-acetylcysteine (NAC), LY294002, SP600125, and KU55933 were purchased from Sigma (St. Louis, MO, USA). Anti-p53 (Cat. no. 2524), anti-phospho-Akt^Thr308^ (Cat. no. 9275S), anti-phospho-Akt^ser473^ (Cat. no. 4060S), anti-JNK (Cat. no. 9258), anti-phospho-JNK (Cat. no. 9251) antibodies, and U0126 were purchased from Cell Signaling Technology, Inc. (Beverly, MA, USA). Anti-PUMA (Cat. no. ab9643) and anti-phospho-ATM (ATM^Ser1981^) kinase (Cat. no. ab78-100) antibodies were procured from Abcam (Cambridge, UK). Anti-Fas (Cat. no. sc-74540), anti-phospho-ERK (Cat. no. sc-7383), anti-phospho-p38 (Cat. no. sc-7937), and anti-*β*-actin (Cat. no. sc-47778) antibodies were purchased from Santa Cruz Biotechnology, Inc. Terminal deoxynucleotidyl transferase-mediated dUTP-fluorescein nick end-labeling (TUNEL) assay kit was purchased from Roche Diagnostics (Mannheim, Germany). JNK-specific siRNA was obtained from Applied Biosystems (Foster City, CA).

### 2.2. Mouse Lung Fibroblast Isolation and Culture

ICR mice aged 8–10 weeks were dissected under asphyxia. The lungs and upper airway were removed and placed into 10 cm Petri dishes containing HSBB buffer. After rinsing twice with HBSS buffer, the tissues were minced into 1–3 mm pieces and incubated with trypsin, collagenase, and DNase. The samples were filtered, and equivalent medium was added to discontinue decomposition. After centrifugation, cells were harvested and cultured in DMEM-10% FBS supplemented with 1% L-glutamine, 1% nonessential amino acid, penicillin (100 units/mL), and streptomycin (100 *μ*g/mL). The viability of isolated lung fibroblasts was approximately 75%. All experiments were performed with primary mouse lung fibroblasts at 3 to 15 passages. Average plating efficiency was >95%.

### 2.3. Western Blot Analysis

Cells were lysed at 4°C in RIPA buffer containing 50 mM Tris-HCl (pH 7.4), 150 mM NaCl, 1% Triton X-100, 0.25% sodium deoxycholate, 5 mM EDTA (pH 8.0), and 1 mM EGTA, and supplemented with protease and phosphatase inhibitors. After 20 min of lysis on ice, cell debris was removed by microcentrifugation, followed by quick freezing of the supernatants. The protein concentration was determined by the Bradford method. Equal amounts of total protein were separated onto SDS-polyacrylamide gels and then electrophoretically transferred from the gel onto a PVDF membrane (Millipore, Bedford, MA). Membranes were blocked for 1 h in phosphate-buffered saline (PBS) containing 0.1% Tween-20 and 10% non-fat dry milk (NFDM). After blocking, the membrane was reacted with specific primary antibodies against p-Akt^ser473^ (1 : 1000), p-Akt^thr308^ (1 : 1000), p-ERK (1 : 1500), p-JNK (1 : 1000), JNK (1 : 1000), p-p38MAPK (1 : 1000), p-ATM (1 : 1000), p53 (1 : 1000), PUMA (1 : 1000), Fas (1 : 1000), and *β*-actin (1 : 2000), overnight at 4°C. Following extensive washings with PBS/T (5 changes over 30 min), membranes were incubated with HRP-conjugated goat anti-rabbit or goat anti-mouse secondary antibodies (1 : 10,000) (PerkinElmer, Inc., Boston, MA) for 1 h. The blots were visualized using an ECL-Plus detection kit (PerkinElmer Life Sciences, Inc. Boston, MA, USA).

### 2.4. Apoptotic Cell Determination

After treatment, cells were fixed with 2% paraformaldehyde for 20 min and washed twice with PBS. Then, the cells were permeabilized with 0.1% Trion-X 100 for 30 min and incubated in a TUNEL reaction buffer for 2 h. TUNEL assay protocol was carried out according to the manufacturer's instructions. After reaction with the TUNEL buffer, cells were incubated with DAPI for 10 min, and the images were visualized using a fluorescence microscope. TUNEL-positive cells were counted as apoptotic cells by flow cytometry.

### 2.5. Measurement of Reactive-Oxygen-Species-(ROS) Generation

Dihydroethidine (DCF-DA) is a specific superoxide tracing dye, which is frequently used to monitor H_2_O_2_ and hydroxyl radical levels in cells. To detect the levels of intracellular ROS production, cells were incubated for the indicated times in the absence or presence of gallic acid and then treated with 5 *μ*M dihydroethidine or 5 *μ*M H_2_DCF-DA (Molecular Probes, Eugene, OR, USA) for 30 min prior to harvesting. After rinsing twice with PBS, cells were detached, and fluorescence was measured with a FACS Calibur flow cytometer using Cell Quest software.

### 2.6. siRNA Transfection

To knockdown JNK expression, synthetic JNK siRNA duplex oligomer and a scrambled siRNA duplex oligomer were purchased from Applied Biosystems. For siRNA transfection experiments, mouse lung fibroblasts were plated onto 60 mm dishes (1 × 10^5^ per dish) and cultured overnight in complete medium. The following morning, cells were transiently transfected with Oligofectamine (Invitrogen) supplemented with JNK siRNA (30 and 50 nM) for 16 h. The levels of specific protein were detected by immunoblotting by treating with gallic acid for indicated times. The apoptotic cells were measured after 24 h gallic acid administration.

### 2.7. Statistical Analysis

All the figures shown in this paper were obtained from at least three independent experiments with similar results. All data are presented as mean ± SD of at least three separate experiments. Statistical differences were evaluated using the Student's *t*-test and considered significant at *P < 0.05, **P < 0.01, or ***P < 0.001.

## 3. Results

### 3.1. Involvement of JNK Activation in Gallic Acid-Mediated Apoptosis

**Figure 1:**
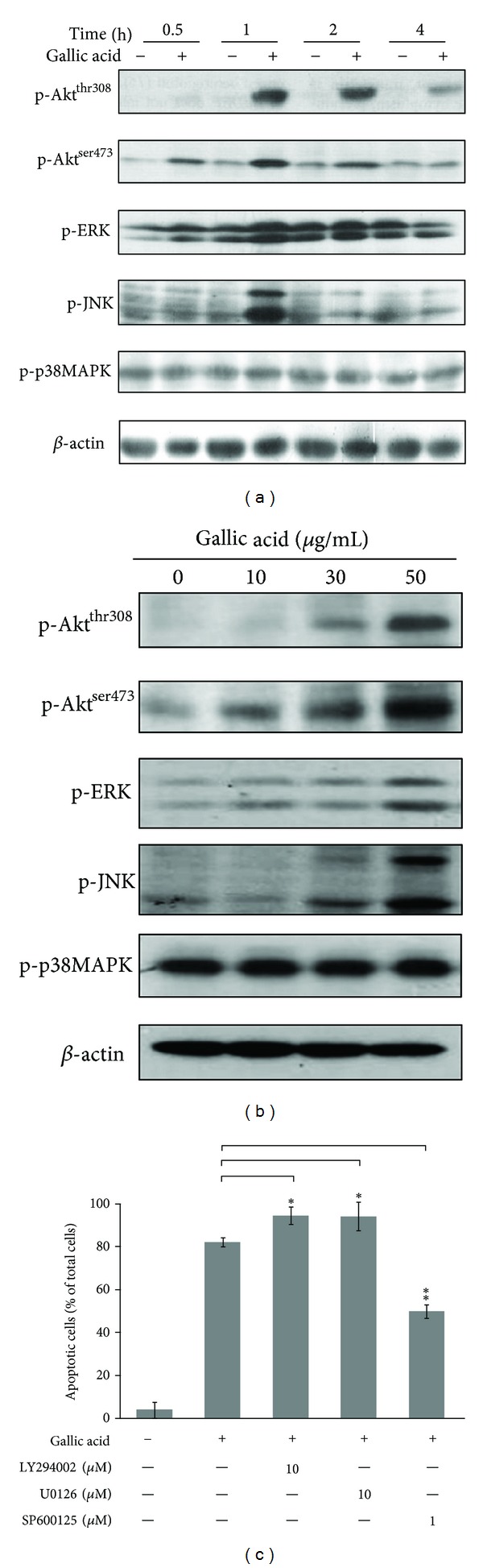
Involvement of JNK activation in gallic acid-mediated apoptosis. (a) Time effect. MLFs were treated with 50 *μ*g/mL gallic acid for 0.5, 1, and 2. (b) Dose effect. MLFs were treated with 0, 10, 30, and 50 *μ*g/mL gallic acid for 1 h. After incubation, the activation of p-Akt, p-ERK, p-JNK, and p-p38MAPK was detected by Western blot analysis. *β*-Actin was used as an internal loading control. (c) MLFs were pretreated in the presence of the specific inhibitors of Akt, ERK, and JNK (LY294002, U0126, and SP600125, respectively) for 1 h and then incubated with 50 *μ*g/mL gallic acid for 24 h. The apoptotic cells were determined by TUNEL assay. Data were expressed as the mean ± SD from 3 independent experiments.

Our previous studies showed that the ROS-mediated ATM/p53 signaling plays a critical role in gallic acid-induced cell death in primary cultured mouse lung fibroblasts. It was found that the inhibition of ATM/p53 activity by pharmacologic and genetic strategies partially blocked the gallic acid-induced apoptotic process [16], indicating that another pathway might also be involved in gallic acid-triggered lung fibroblast apoptosis. It has also been reported that mitogen-activated protein kinase (MAPK) and phosphoinositide 3-kinase (PI3K)/protein kinase B (PKB/Akt) signaling pathways are the primary intermediates for the induction of apoptosis by oxidative stress [19]. Our recent report demonstrated that gallic acid-induced ROS generation and apoptotic cell death is in a time- and dose-dependent manner [16]. Thus, the time and dose effect of gallic acid on the activity of MAPKs and Akt in mouse lung fibroblasts was examined by immunoblot analysis using antibodies against phosphorylated form of MAPKs and Akt. In this study, we found that gallic acid exerts time- and dose-dependent effects in levels of phosphorylated JNK, ERK, and Akt in lung fibroblasts (Figures 1(a) and 1(b)). However, no visible p38MAPK phosphorylation was observed. The total amounts of ERK, JNK, p38MAPK, and Akt were not affected by gallic acid (data not shown). 

To address the potential role of Akt, ERK, and JNK phosphorylation in gallic acid-induced apoptosis, mouse lung fibroblasts were exposed to gallic acid in the presence of specific inhibitors of Akt, ERK, and JNK (LY294002, U0126, and SP600125, respectively). The percentage of gallic acid-induced apoptotic cells was then determined by TUNEL assay at 24 h. As shown in Figure 1(c), gallic acid-induced apoptosis was significantly inhibited by pretreatment of SP600125. In contrast, pretreatment with LY294002 and U0126 accelerated gallic acid-mediated apoptosis in mouse lung fibroblasts. These results revealed that activation of JNK is mostly involved in gallic acid-induced apoptotic cell death. However, activation of ERK and Akt may protect mouse lung fibroblasts against gallic acid-mediated cell death.

### 3.2. JNK Activation Contributes to Gallic Acid-Elicited p53 Activation, Fas and PUMA Expression, and Apoptosis Induction

**Figure 2:**
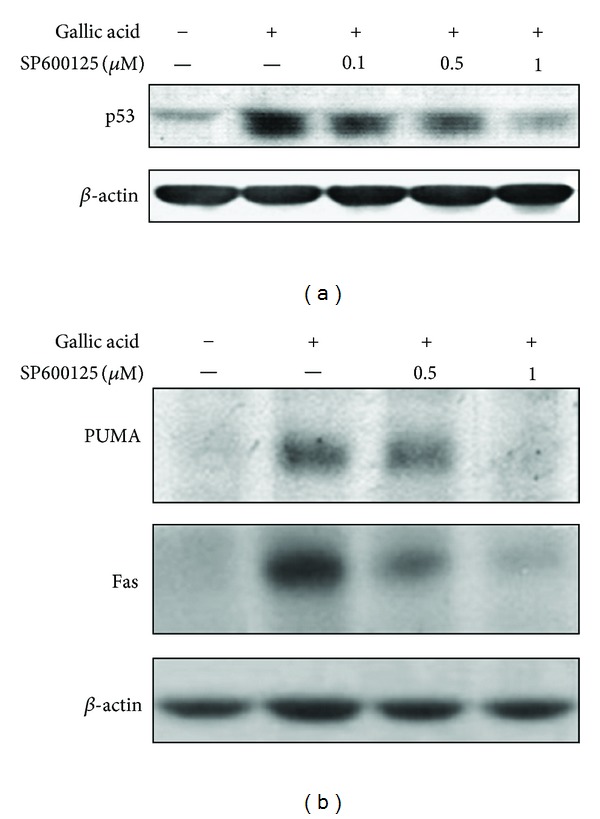
Involvement of JNK in gallic acid-elicited p53 accumulation and apoptosis-related molecule expression. (a) MLFs were pretreated with the indicated concentrations of SP600125 for 1 h and then incubated with 50 *μ*g/mL gallic acid for 1 h. Cell lysates were analyzed by Western blot with antibodies against p53. (b) MLFs were pretreated with SP600125 for 1 h and then incubated with 50 *μ*g/mL gallic acid for 24 h. Cell lysates were analyzed by Western blot with antibodies against PUMA and Fas. *β*-Actin was used as an internal loading control.

JNK has been shown to activate p53 in response to different stressful stimuli, and such phosphorylation can initiate p53 response, leading to cell cycle arrest and apoptosis [20]. To examine whether JNK activation plays a role in gallic acid-induced p53 accumulation and downstream apoptotic events, mouse lung fibroblasts were pretreated with SP600125 (0.1, 0.5, and 1 *μ*M) for 1 h prior to gallic acid incubation. The levels of p53, PUMA, and Fas were determined by Western blotting. Consistent with the results of previous studies, exposure to gallic acid significantly increased the levels of p53 (Figure 2(a)); however, pretreatment with JNK inhibitor SP600125 dose dependently reduced p53 levels. Similarly, gallic acid-mediated increase of proapoptotic proteins, PUMA and Fas protein levels, was also attenuated by pretreatment with SP600125 (Figure 2(b)). 

**Figure 3:**
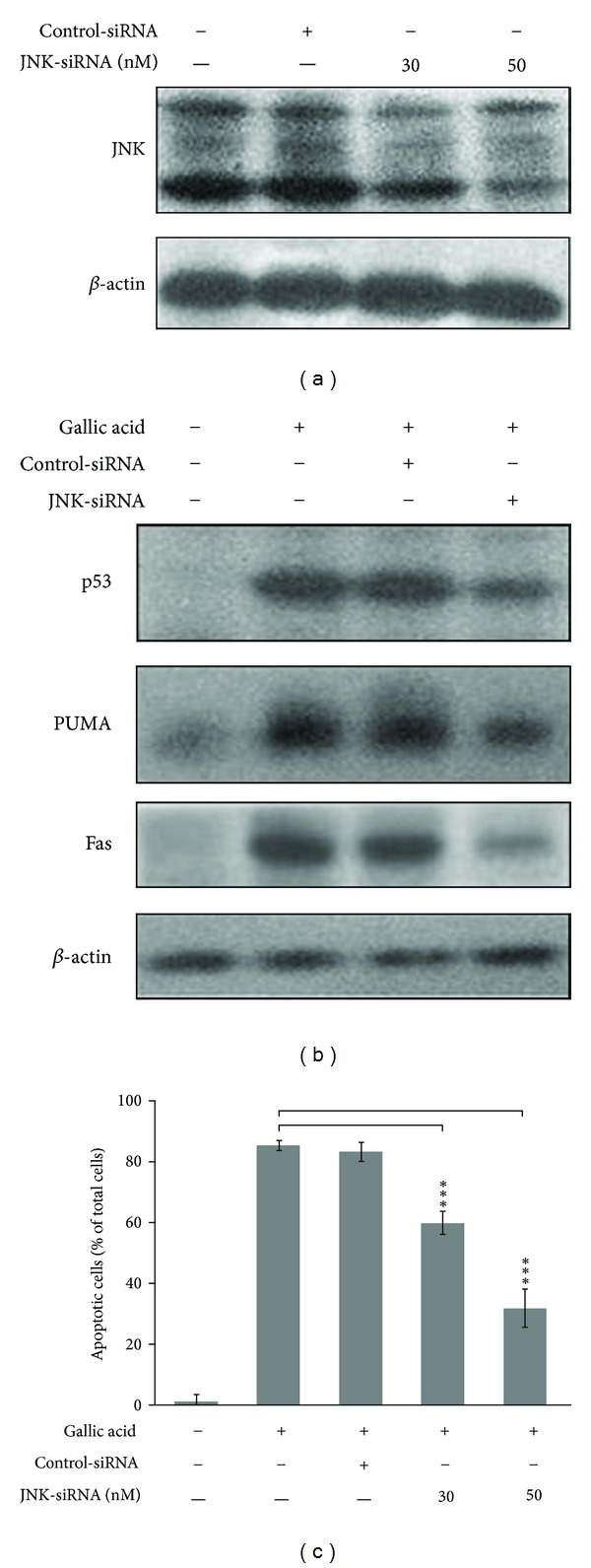
Knockdown of JNK prohibited the upregulation of gallic acid-elicited p53 accumulation and apoptosis-related molecule expression. (a) MLFs were treated with control-siRNA or the indicated concentrations of JNK siRNA for 16 h. Cell lysates were analyzed by Western blot with antibodies against JNK. (b) MLFs were treated with control-siRNA or JNK siRNA in maintenance medium for 16 h followed by stimulation with 50 *μ*g/mL gallic acid for 1 h (for p53) or 24 h (for PUMA and Fas). Cell lysates were analyzed by Western blot with antibodies against p53, PUMA, and Fas. (c) MLFs were treated with control-siRNA or JNK siRNA in maintenance medium for 16 h followed by stimulation with 50 *μ*g/mL gallic acid for 24 h. The apoptotic cells were determined by TUNEL assay. Data were expressed as the mean ± SD from three independent experiments.

To further confirm the role of JNK in gallic acid-triggered p53 accumulation, Fas and PUMA expression, and to avoid nonspecific effects of SP600125, knockdown of JNK expression by JNK-specific siRNA in mouse lung fibroblasts was carried out. As expected, the level of JNK was suppressed by JNK siRNA in a dose-dependent manner (Figure 3(a)). Gallic acid-induced Fas and PUMA upregulation (Figure 3(b)) and cytotoxicity (demonstrated by decrease in TUNEL-positive cells) were also diminished in JNK-siRNA-treated mouse lung fibroblasts, compared with control-siRNA treated culture (Figure 3(c)). These results indicated that JNK plays an upstream role in the gallic acid-induced p53 activation and apoptotic signaling pathway. 

### 3.3. Gallic Acid-Provoked ROS Generation Is Required for JNK Activation and Downstream Apoptotic Process

**Figure 4:**
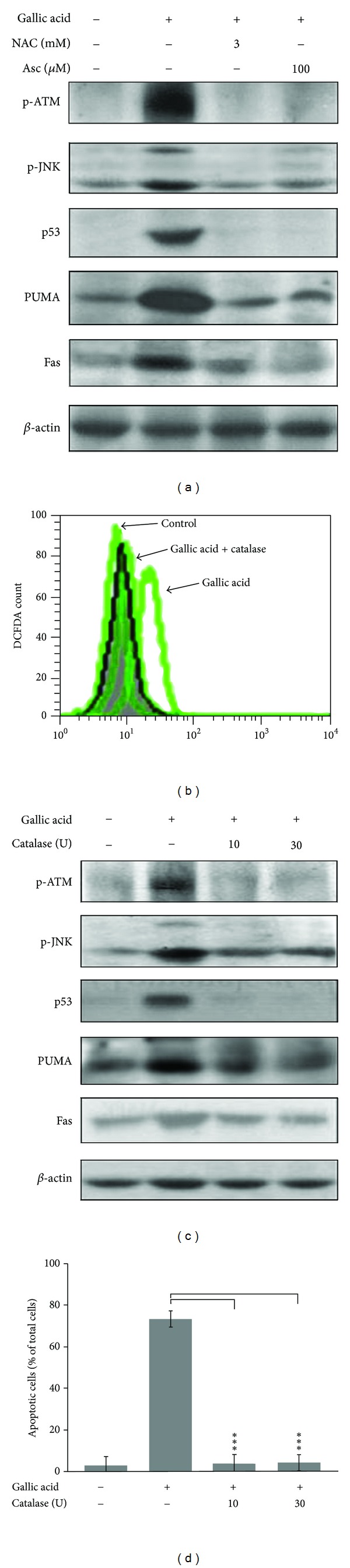
Antioxidants and catalase inhibit gallic acid-mediated ROS production and apoptotic process. (a) MLFs were pretreated with 100 *μ*M ascorbic acid (Asc) or 3 mM N-acetylcysteine (NAC) for 30 min and then incubated with 50 *μ*g/mL gallic acid for another 1 h or 24 h. The levels of p-ATM, p-JNK, p53, PUMA, and Fas were detected by Western blot analysis. (b)-(c) MLFs were preincubated with catalase for 1 h and then treated with gallic acid for another 30 min for DCF-DA fluorescence analysis by flow cytometry or 24 h for apoptosis determination by TUNEL assay. (d) MLFs were preincubated with catalase for 1 h and then treated with gallic acid for another 1 h. The levels of p-ATM, p-JNK, p53, PUMA, and Fas were detected by Western blot analysis.

To examine whether JNK signaling pathway is also required for gallic acid response through ROS production, mouse lung fibroblasts were exposed to gallic acid in the absence or presence of antioxidants, N-acetylcysteine (NAC), and ascorbic acid (ASC). The levels of phosphorylated JNK, p53, PUMA, and Fas were determined by Western blot. As expected, antioxidants (both ASC and NAC) significantly abolished the gallic acid-induced JNK and p53 activation as well as PUMA and Fas upregulation (Figure 4(a)), suggesting that ROS induced by gallic acid plays a crucial role in JNK phosphorylation and proapoptotic protein expression in lung fibroblasts. Our previous report suggested that the relative level of hydrogen peroxide was elevated at 30 min after gallic acid treatment [16]. To obtain further insight into the effects of catalase, an antioxidative enzyme, on the gallic acid-mediated hydrogen peroxide production and apoptotic process, mouse lung fibroblasts were preincubated with catalase for 1 h and then treated with gallic acid for another 30 min (for ROS generation assay) or 24 h (for apoptosis determination). As shown in Figure 4(b), the addition of catalase completely inhibited hydrogen peroxide formation of mouse lung fibroblasts. Moreover, catalase treatment effectively inhibited the phosphorylation of ATM and JNK. This event was accompanied by decreased expression of p53, PUMA, and Fas (Figure 4(c)), as well as mouse lung fibroblast apoptosis (Figure 4(d)). These data revealed that gallic acid-mediated hydrogen peroxide formation acts as an upstream regulator of ATM, JNK, and p53 activation and Fas and PUMA upregulation, to exert its apoptotic influence in mouse lung fibroblasts.

### 3.4. Synergistic Effect of ATM and JNK on Gallic Acid-Induced Mouse Lung Fibroblasts Apoptosis

**Figure 5:**
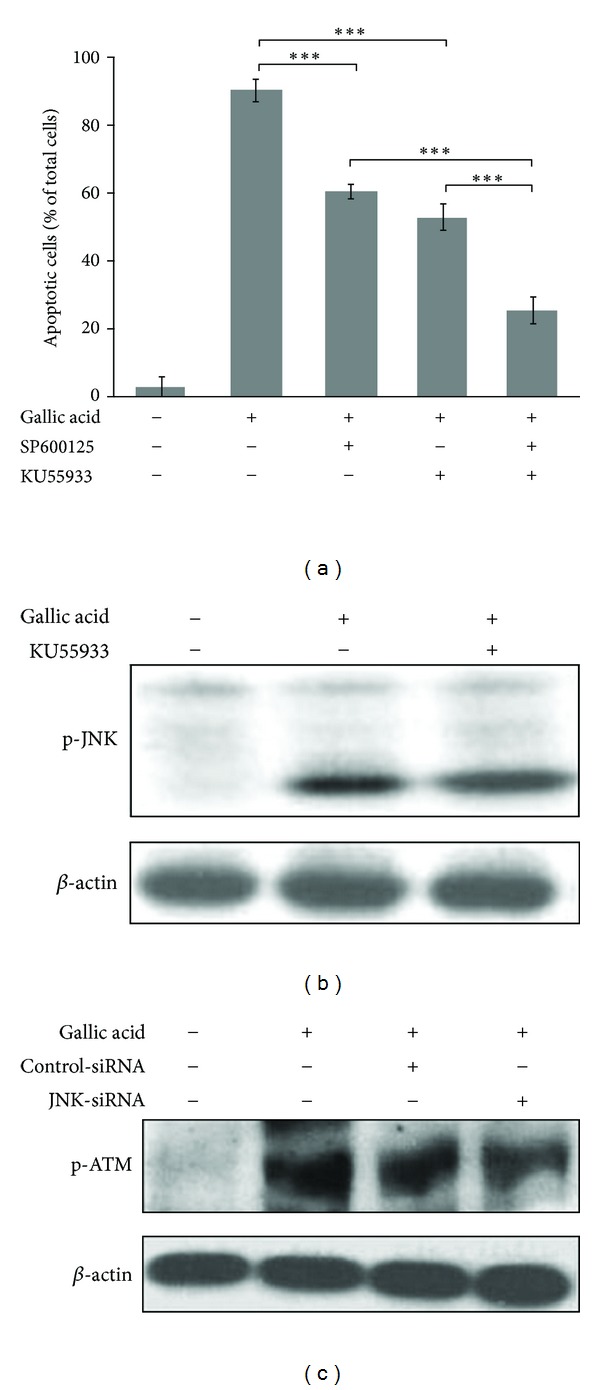
Synergistic effects of ATM and JNK on gallic acid-induced MLF apoptosis. (a) MLFs were pretreated with SP600125 and/or KU55933 for 1 h and then incubated with 50 *μ*g/mL gallic acid for 24 h. The apoptotic cells were determined by TUNEL assay. Data were expressed as the mean ± SD from 3 independent experiments. (b) MLFs were pretreated with KU55933 (ATM kinase inhibitor) for 1 h and then incubated with 50 *μ*g/mL gallic acid for another 1 h. Cell lysates were analyzed by Western blot with antibodies against p-JNK. (c) MLFs were pretreated with SP600125 (JNK kinase inhibitor) for 1 h and then incubated with 50 *μ*g/mL gallic acid for another 1 h. Cell lysates were analyzed by Western blot with antibodies against p-ATM.

Based on other's and our studies, both ATM and JNK are upstream regulators of p53 phosphorylated activation [16, 21]. To characterize the interplay between ATM and JNK during gallic acid-mediated apoptotic process, mouse lung fibroblasts cells were treated with ATM kinase inhibitor KU-55933 and/or JNK inhibitor SP600125 prior to addition of gallic acid. As shown in Figure 5, pretreatment of KU-55933 or SP600125 alone only partially diminished gallic acid-mediated cytotoxicity, as demonstrated by a decrease in TUNEL-positive cells. However, a treatment with both KU-55933 and SP600125 displayed a synergistic protection of mouse lung fibroblasts against gallic acid-elicited apoptosis. To explore the interplay between ATM and JNK in gallic acid-induced apoptosis, the effect of ATM inhibitor on the JNK phosphorylation was examined. As shown in Figure 5(b), pretreatment of ATM inhibitor KU-55933 did not affect gallic acid-induced phosphorylation of JNK. Next, the influence of JNK inhibition on ATM phosphorylated activation was also investigated. As indicated in Figure 5(c), inhibition of JNK activity by SP600125 could alter the levels of phosphorylated ATM induced by gallic acid (Figure 5(c)). Our data suggested that ATM and JNK contribute to two different pathways with synergistic effect on gallic acid-triggered mouse lung fibroblast apoptosis. 

## 4. Discussion

Idiopathic pulmonary fibrosis is a progressive interstitial lung disorder with no effective therapies. There is growing evidence demonstrating that the activation of pulmonary fibroblast is a key issue in the pathogenesis of lung fibrosis. Therefore, recent antifibrotic treatment has focused on the inhibition of lung fibroblasts activation and its related subsequent events, such as extracellular matrix deposition and enhanced proliferation [22]. Antioxidative agents are useful in both the prevention of lung injury and the attenuation of fibrogenesis, and many agents exhibit their antifibrotic effects through this mechanism [23]. Gallic acid is a natural phenolic compound with strong antioxidative activity [24]. Our previous study showed that gallic acid induces apoptosis in mouse lung fibroblasts. Treatment with gallic acid activates ROS-mediated DNA damage signaling pathway by triggering ATM-dependent activation of p53. The transcriptional activation of p53 upregulates the proapoptotic molecules, such as PUMA and Fas, and provokes caspase activation via both intrinsic and extrinsic pathways, consequently leading to apoptotic cell death [16]. However, treatment with ATM inhibitor cannot completely block gallic acid-induced p53 activation and cell death, suggesting that another pathway may be involved in p53 activation and subsequent gallic acid-mediated cytotoxic effect. In this study, we aimed to examine new insights into the other possible mechanisms of gallic acid-induced apoptosis in mouse lung fibroblasts. Our observations showed that JNK activation also contributes to gallic acid-elicited p53 activation and apoptosis induction. Gallic acid-mediated increases of proapoptotic proteins, PUMA and Fas protein levels, are attenuated by pharmacological and genetic inhibition (JNK inhibitor SP600125 and JNK-specific siRNA) of JNK. Moreover, a treatment with both ATM and JNK inhibitor (KU-55933 and SP600125, respectively) displays a synergistic protection of mouse lung fibroblasts against gallic acid-elicited apoptosis. These findings reveal that JNK-dependent p53 activation is another pathway involved in gallic acid-induced apoptosis. 

Gallic acid, commonly distributed in various plants, fruits, and foods [24], has anticancer activity and induces apoptotic cell death in various types of cancer cells, such as prostate [25], lung [26, 27], gastric, colon, breast, cervical, and esophageal [28]. There is increasing evidence suggesting that apoptosis induced by gallic acid is associated with oxidative stress derived from reactive-oxygen-species-(ROS), mitochondrial dysfunction, and an increase in intracellular Ca^2+^ level [29–31]. Inoue et al. reported that the intracellular peroxide level induced by gallic acid in HL-60RG cells was well correlated with the potency to induce apoptosis, and that the increased intracellular peroxides after gallic acid treatment seemed likely to have resulted from the influx of H_2_O_2_, which was generated extracellularly [32]. Similarly, we found that pretreatment with catalase completely inhibited gallic acid-triggered hydrogen peroxide accumulation and apoptotic death in lung fibroblasts (Figures 4(b) and 4(c)). Moreover, pretreatment with antioxidants, ascorbic acid, and NAC, as well as catalase significantly attenuated gallic acid-elicited ATM, JNK, and p53 activation, and subsequently increased PUMA and Fas protein levels (Figures 4(a) and 4(d)). These results suggested that hydrogen peroxide induced by gallic acid acts as an upstream signal that stimulates the activation of both ATM and JNK and then induces a p53-dependent apoptosis in lung fibroblasts.

In cells, numerous stress response signaling molecules are rapidly activated in response to oxidative insults. Some of these molecules are preferentially linked to enhanced survival, while others are more frequently associated with cell death. Mitogen-activated protein kinases (MAPKs), including extracellular signal regulated kinase (ERK1/2), c-Jun N-terminal kinase/stress-activated protein kinase (JNK/SAPK), and p38MAPK, are involved in cell proliferation and differentiation and cell death [33]. There is growing evidence indicating that ROS can stimulate the activation of ERK [34, 35], JNK, and p38MAPK [36–38]. In most instances, ERK activation has a prosurvival function, rather than proapoptotic effects [39]. Several studies demonstrate that ERK activation serves as a survival factor following oxidant injury; inhibition of ERK activation sensitizes cells to hydrogen peroxide [40]. Consistent with this study, exposure to gallic acid increased the levels of phosphorylated ERK (p-ERK) (Figure 1(a)). Treatment with ERK inhibitors (U0126) accelerated gallic acid-mediated apoptosis in mouse lung fibroblasts (Figure 1(b)), suggesting that activation of ERK might act as a prosurvival factor in this event. Akt, known as protein kinase B, is a serine/threonine kinase which is activated via a phosphoinositide 3-kinase (PI3K) pathway [41]. Like ERK, Akt is also an important antiapoptotic prosurvival kinase during the cellular response to oxidant injury [42]. Sonoda et al. reported that administration of cells with wortmannin blocked hydrogen peroxide-induced Akt activation and increased cell death [43]. Using a genetic approach to elevate Akt expression directly supports the evidence that Akt plays an important role in enhancing cell survival following oxidant injury in hydrogen peroxide-treated HeLa and NIH3T3 cells [44]. In the results of this study, we also found that activation of Akt was accompanied by gallic acid-provoked ROS generation; however, treatment with LY294002 to inactivate Akt significantly accelerated gallic acid-induced cell death. These results suggest that activation of ERK and Akt is possibly increased as a result of intracellular ROS stress that further induces anti-apoptotic signaling to protect cell against oxidative injury upon gallic acid treatment. 

The JNK and p38MAPK pathways are noted for their activation by a wide range of stresses including cytokines, radiation, osmotic shock, mechanical injury, heat stress, and oxidative damage [45]. In general, the activation of JNK and p38MAPK by ROS leads to apoptosis in various types of cells [36–38]. The JNK inhibitor could protect rat pheochromocytoma PC12 cells against gallic acid-triggered cell death [46], while the p38MAPK inhibitor was found to decrease the death induced by pyrogallol in calf pulmonary artery endothelial cells [47]. Here, we provide evidence that ROS-mediated JNK activation, but not p38MAPK, is an early regulator in response to gallic acid treatment, which occurs concomitantly with the onset of apoptosis. Treatment with the chemical JNK inhibitor SP600125 and JNK-specific siRNA significantly attenuated apoptosis following gallic acid treatment (Figures 1(b), 2, and 3), suggesting that the ROS-induced JNK activation plays an important role in the apoptosis of mouse lung fibroblasts. However, Park reported that both JNK and p38 inhibitors did not affect cell death, ROS, and GSH levels in the gallic acid-treated human pulmonary fibroblast cells [48]. It is possible that the anti- or proapoptotic effects of the MAPKs by ROS on gallic acid-treated cells may vary depending on cell type and treated conditions.

The tumor suppressor protein p53 constitutes a potential target of proapoptotic signaling by JNK and exerts a proapoptotic influence in response to oxidative stress. It has been reported that p-JNK physically interacts with p53 and stabilizes it by phosphorylation at residue threonine 81. The phosphorylation of p53 at threonine 81 is required for the dissociation of p53 from Ubc13, leading to p53 accumulation, multimerization, and transcriptional activation [49]. Stress and damage stimuli-triggered apoptosis has been shown to be induced through activation of p53 *via *JNK signaling in HRas MCF10A cells [50], Lewis lung carcinoma (LLC) cells, hepatoma HepG2 cells, and Molt-4 leukemia cells [51]. Silibinin, a mixture of flavonolignans, induces p53-mediated cell death via ROS-mediated JNK activated pathways in human cervical carcinoma HeLa cells [52] and in human fibrosarcoma HT1080 cells [53]. Our current study showed that ROS-mediated JNK activation was accompanied by p53 activation. Pharmacological and genetic inhibition of JNK by SP600125 and JNK-specific siRNA effectively abolished p53 accumulation and PUMA/Fas expression, indicating that gallic acid-induced apoptosis occurs via ROS-JNK-p53-PUMA/Fas signaling pathway. 

**Figure 6:**
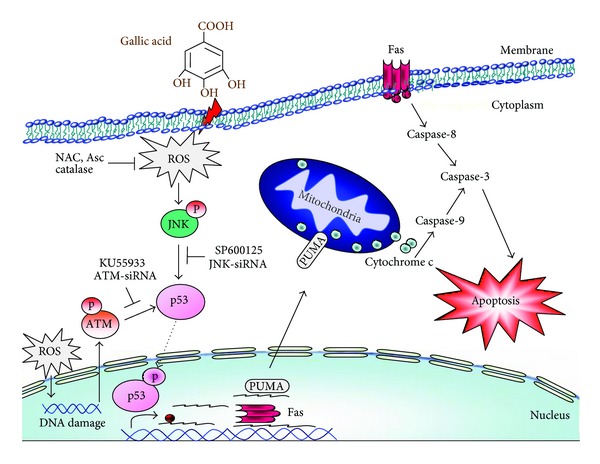
Schematic model of gallic acid-induced apoptosis pathway in primary cultured murine lung fibroblasts. Incubation of fibroblasts with gallic acid activated ROS-mediated DNA damage signaling pathway by triggering both JNK and ATM-dependent activation of p53. The transcriptional activation of p53 upregulated the proapoptotic molecules, such as PUMA and Fas, consequently leading to apoptotic cell death.

In conclusion, our previous studies revealed that ROS-mediated ATM activation is an upstream regulator of p53 activation in gallic acid-induced cell death in mouse lung fibroblasts [16]. Here, we provide evidence that ROS-induced JNK activation is an initiator that mediates p53 accumulation and activation and the subsequent increase of proapoptotic protein PUMA and Fas expression. Based on our previous study, as well as the present study, it is apparent that gallic acid most likely exerts its antifibrotic effects directly through the ROS-JNK/ATM-p53 signaling pathways, utilizing both mitochondria (PUMA) and death receptor (Fas) as the effectors of cell death (Figure 6). Gallic acid has been studied in vivo exhibiting antiproliferative, proapoptotic, and antitumorigenic effects in xenograft animal models [25, 54, 55]. Furthermore, gallic acid treatment has been also shown to induce apoptosis of rheumatoid arthritis fibroblast-like synoviocytes isolated from patients [56]. Our data provide the molecular mechanisms of gallic acid in the fight against lung fibroblasts in an in vitro model. However, the in vivo animal model study should be performed for further evaluating the possible application of this compound in the prevention and perhaps in therapy for pulmonary fibrosis.
